# Soft tissue recurrence in giant cell tumor of Bone: A comprehensive review of pathogenesis, imaging features, and clinical management

**DOI:** 10.1016/j.jbo.2025.100725

**Published:** 2025-11-08

**Authors:** Khodamorad Jamshidi, Hamed Naghizadeh, Sadegh Saberi, Farshad Zand Rahimi, Aidin Arabzadeh, Seyyed Saeed Khabiri

**Affiliations:** a Bone and Joint Reconstruction Research Center, Department of Orthopedics, School of Medicine, Iran University of Medical Sciences, Tehran, Iran; b Joint Reconstruction Research Center, Tehran University of Medical Sciences, Tehran, Iran; c Department of Orthopedic Surgery, Imam Khomeini Hospital Complex, Tehran University of Medical Sciences, Tehran, Iran

**Keywords:** Giant cell tumor of bone, Soft-tissue recurrence, Pathogenesis, Imaging, Surgical management, Outcomes

## Abstract

**Background:**

Giant cell tumor of bone (GCTB) is a benign but locally aggressive neoplasm with a high risk of recurrence. Among its patterns of relapse, soft-tissue recurrence (STR) is an uncommon but clinically significant entity, often presenting as ossified or non-ossified perilesional nodules. Despite its rarity, STR poses diagnostic and therapeutic challenges that require clarification.

**Methods:**

A comprehensive literature review was performed across PubMed, Embase, and Google Scholar from 1980 through January 2025, focusing on the epidemiology, pathogenesis, imaging features, histopathology, management, and outcomes of STR in GCTB. Case reports, series, and retrospective studies explicitly distinguishing STR from intraosseous recurrence were included, and findings were synthesized narratively.

**Results:**

STR occurs in approximately 2–3 % of GCTB cases, typically within 6–12 months after surgery. Major risk factors include curettage procedures, pathological fractures, cortical breaches, and unrecognized microscopic soft-tissue extension. Imaging reveals three distinct patterns: peripheral “eggshell” ossification, central nodular calcification, and purely soft-tissue lesions. Histology mirrors primary GCTB, often with osteogenic metaplasia, while molecular testing confirms retention of H3F3A mutations. Surgical excision with clear margins remains the mainstay of treatment, yielding excellent functional outcomes. However, up to 60 % of patients experience multiple recurrences, highlighting the need for vigilant surveillance. Systemic agents such as denosumab or bisphosphonates remain investigational, and radiotherapy is generally contraindicated due to malignant transformation risk.

**Conclusion:**

STR represents a rare but distinct subset of GCTB recurrences. Awareness of risk factors, early imaging-based detection, and complete surgical excision are critical for optimal outcomes. Further multicenter studies are required to define surveillance protocols, validate molecular predictors, and clarify the role of systemic therapy in this challenging condition.

## Highlights

• Soft-tissue recurrence (STR) occurs in 2–3% of giant cell tumors of bone (GCTB), usually within the first postoperative year.
• Major risk factors include curettage procedures, cortical breaches, and pathological fractures, which facilitate tumor cell seeding.
• Imaging reveals three characteristic patterns: peripheral “eggshell” ossification, central nodular calcification, and purely soft-tissue lesions.
• Histology and H3F3A mutation status in STR mirror primary GCTB, supporting its nature as a true recurrence.
• Complete surgical excision with negative margins remains the mainstay of treatment, ensuring excellent functional outcomes.
• Up to 60 % of patients experience multiple STRs, underscoring the need for close surveillance during the first 24 months.
• Systemic therapies (denosumab, bisphosphonates) may be used off-label in selected cases, and radiotherapy is contraindicated due to malignant transformation risk.

## 1 Introduction

Giant cell tumors of bone (GCTB) represent approximately 5 % of primary bone tumors and typically affect the epiphyses of long bones in young adults [1]. Despite advances in surgical techniques, local recurrence remains a notable challenge, occurring in 10–30 % of cases [2]. Among these recurrences, soft tissue recurrence (STR) constitutes a distinct entity characterized by tumor regrowth limited to perilesional soft tissues without osseous involvement [3].

STR typically manifests as a painless mass near the surgical site, emerging months to years after the initial treatment [4]. Imaging studies reveal three distinct patterns: peripheral “eggshell” ossification, central nodular ossification, and purely soft-tissue nodules [5,6]. Current evidence suggests an association between STR development and several factors, including intraoperative tumor seeding, residual soft tissue disease, and hematoma contamination from pathological fractures [4,7]. Although extended curettage often achieves local control, the optimal surveillance strategy and role of adjuvant therapies remain uncertain [8,9].

This review addresses five critical questions regarding STR in GCTB: (1) What is the true prevalence, and what factors increase the risk? (2) Which imaging features enable early detection? (3) Are there distinct histopathological or molecular characteristics? (4) What defines optimal surgical management and *peri*-operative adjuvant strategies, excluding systemic adjuvant therapy? (5) What are the functional and survival outcomes following surgical treatment of STR? Clarifying these issues will guide evidence-based management and inform future research.

## 2 Methods

We conducted a comprehensive literature search across PubMed, Embase, and Google Scholar, including studies published from 1980 through January 2025. Search terms included “giant cell tumor of bone,” “soft tissue recurrence,” “local recurrence,” “pathogenesis,” “imaging,” and “management.” We manually reviewed the reference lists to identify additional relevant publications.

Studies were included if they explicitly distinguished STR from intraosseous recurrence and provided original data on the incidence, imaging characteristics, histopathology, treatment, or outcomes. Given STR's rarity of STR, we included case reports, case series, and retrospective cohort studies. We excluded review articles lacking primary data, editorials, and studies without clear STR definitions from the study.

Given the predominance of Level IV evidence, a thematic synthesis approach was applied in accordance with the Realist and Meta-narrative Evidence Syntheses (RAMESES) framework [10], allowing for qualitative exploration of recurrent clinical patterns and mechanisms. The findings are presented narratively with illustrative clinical examples to highlight the key management principles.

### 2.1 Epidemiology and risk factors

Contemporary studies indicate that STR occurs in approximately 2.1–2.5 % of GCTB cases. Xu et al. identified eight STR cases among 381 treated lesions (2.1 %) [3]. This figure may underestimate the true incidence, as non-ossified recurrences can escape detection on plain radiographs. When MRI or ultrasound surveillance is systematically employed, the incidence approaches 2–3 % [6,11]. STR typically presents 11.3 ± 4.1 months after surgery (range 5–17 months), with over 60 % occurring within the first year [3,12].This temporal clustering emphasizes the importance of vigilant surveillance using advanced imaging modalities during the initial 6–12 months post-treatment.

Several factors are associated with an increased STR risk. Intralesional curettage, even with chemical adjuvants, correlates with higher STR rates than en bloc resection. In one cohort, 76 % of STR cases underwent curettage procedures [13,14]. Pathological fractures at presentation show a three- to four-fold association with STR development, likely due to tumor cell dissemination through fracture hematomas [4,13].

Anatomic location also influences the occurrence of STRs. Distal radius lesions are disproportionately represented among STR cases [15,16]. While comprising only 10 % of one study population, distal radius tumors accounted for 25 % of STR events [17]. This association may reflect the anatomical constraints and thin soft tissue coverage at this site. Proximal and distal femur locations show similar trends, possibly due to challenges in achieving wide margins [4]. Microscopic soft tissue extension, when present, increases the STR risk if inadequately addressed during surgery [6,13].

**Fig. 1:**
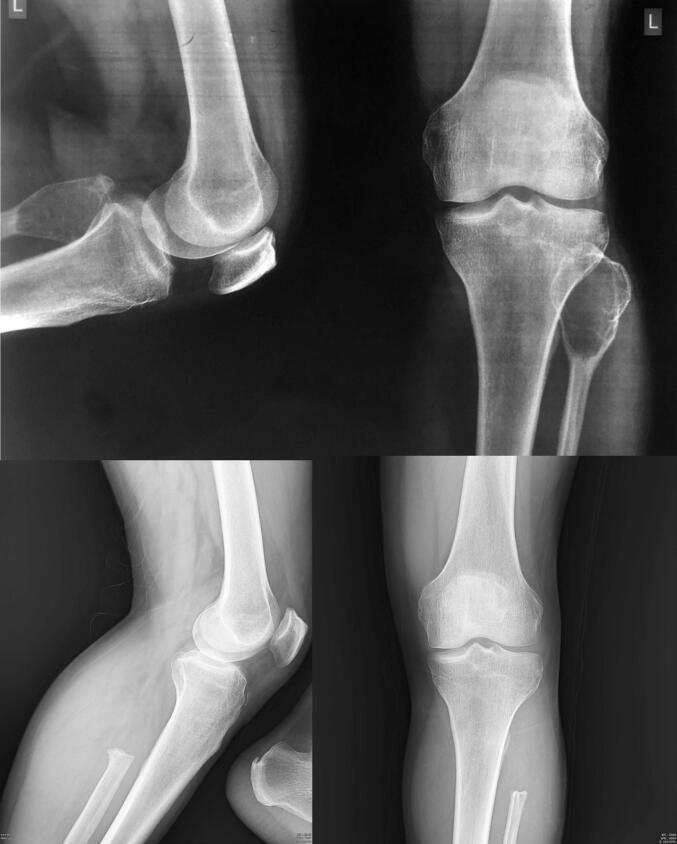
Plain radiographs of the knee in a young male presenting with pain and a palpable lateral proximal leg mass. Images demonstrate a lytic, expansile lesion involving the proximal fibula with cortical thinning and expansion, consistent with a diagnosis of giant cell tumor (GCT), subsequently managed by surgical resection.

Notably, Campanacci grade and tumor volume have not shown independent associations with STR after controlling for the surgical approach and fracture status [3]. The primary modifiable risk factors remain surgical technique, fracture management, and thoroughness of soft tissue clearance [13,[18], [19], [20]] [ Fig. 1].

### 2.2 Proposed mechanisms

Multiple mechanisms contribute to STR development, often acting in synergy. Iatrogenic seeding during intralesional curettage represents the most commonly implicated pathway [4,7,21,22]. Mechanical disruption through curettage, irrigation, or power burr use can displace viable tumor cells into the surrounding tissues [23,24].This risk increases substantially when cortical breaching occurs, whether due to tumor erosion, fracture, or surgical manipulation [25,26].

Pathological fractures create additional contamination pathways for infection. Fracture-associated hematomas allow tumor cells to infiltrate soft tissues beyond the bone surface. If these hematomas or reactive pseudocapsules remain after surgery, embedded tumor cells may proliferate [4]. This mechanism explains the temporal pattern of STR, which typically manifests within the first postoperative year [27,28].

Undetected microscopic soft tissue invasion is another important mechanism. While GCTB primarily affects the bone, subtle extraosseous extension can occur, particularly in larger lesions [29]. High-resolution MRI has revealed small soft tissue foci in patients who later developed STR [30]. These microscopic extensions, if unrecognized during surgery, may persist and manifest as delayed recurrence [6].

Local adjuvant therapies have inherent limitations that may contribute to STR development. Chemical agents such as phenol or alcohol and thermal modalities penetrate only 2–3 mm beyond the application sites [31,32]. When cortical defects are present, escaped tumor cells remain beyond the reach of these adjuvants. This limitation underscores why adjuvant therapy alone cannot prevent STR without adequate mechanical tumor clearance [9,25,33].

These mechanisms often coexist. For example, a patient with a pathological fracture undergoing curettage faces risks from both iatrogenic seeding and hematogenous spread [4,13]. Understanding these overlapping pathways informs prevention strategies focused on minimizing contamination, excising suspicious tissues, and adapting techniques based on individual risk factors [34].

### 2.3 Imaging characteristics

STR demonstrates distinctive imaging patterns that are crucial for diagnosis and management planning. Plain radiographs typically show soft-tissue masses adjacent to previous surgical sites, often with characteristic ossification patterns [3,35]. Peripheral “eggshell” calcification, when present, strongly suggests STR and reflects reactive ossification around implanted tumor cells [4].

Computed tomography provides superior delineation of ossified components and anatomical relationships [6]. A recent classification system categorizes STR into three types based on CT findings: Type I with peripheral rim ossification, Type II featuring central nodular ossification, and Type III without ossification [3]. This classification aids in surgical planning and predicting outcomes.

**Fig. 2:**
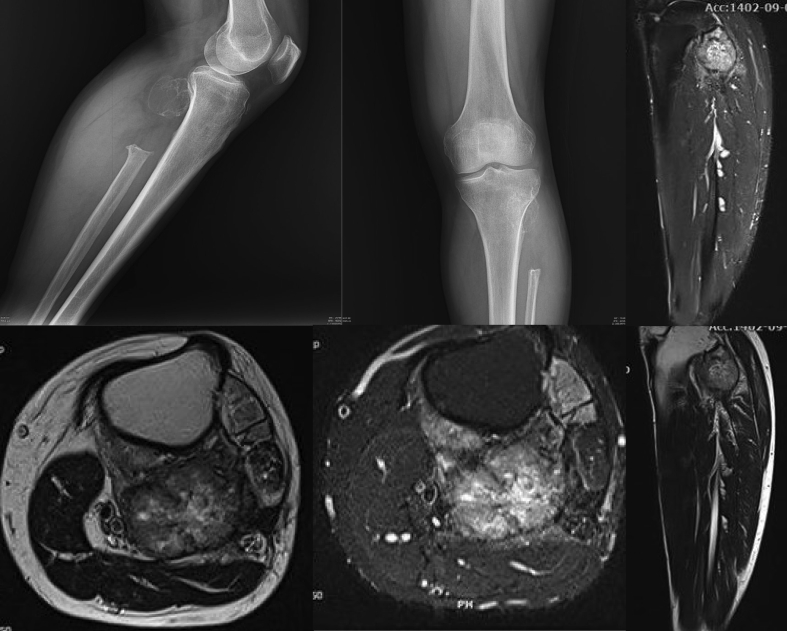
Follow-up imaging one year after resection of proximal fibular giant cell tumor. Radiographs demonstrate a new lytic lesion suggestive of recurrence. MRI sequences show a heterogeneously hypointense mass on T1-weighted imaging and a corresponding hyperintense lesion on T2-weighted imaging, consistent with recurrent GCT at the previous surgical site.

Magnetic resonance imaging remains essential for evaluating non-ossified lesions. Type III STR typically shows intermediate T1 and high T2 signal intensities with heterogeneous enhancement [30]. MRI accurately defines the lesion extent and its relationship to neurovascular structures, which is critical information for surgical planning. Careful differentiation from postoperative changes or aggressive soft tissue neoplasms requires correlation with the clinical history and serial imaging [Fig. 2].

Ultrasound has emerged as a valuable surveillance tool, particularly for superficial lesions. STR appears as hypoechoic, vascularized masses, sometimes with internal calcifications corresponding to ossified components [6]. The accessibility and real-time nature of ultrasound make it ideal for routine follow-up, especially during the high-risk postoperative year [36].

Differentiating STR from postoperative heterotopic ossification, scar tissue, and other benign soft tissue processes remains a challenge. STR typically presents as distinct soft tissue masses with progressive growth noted on serial imaging, whereas heterotopic ossification tends to stabilize and mature early postoperatively, demonstrating a characteristic zonal maturation pattern [37,38].

### 2.4 Timing and diagnostic Clues

Most STR cases present within 6–12 months after surgery, although detection may occur up to 30 months postoperatively [6,27]. The mean detection interval of 11.3 months emphasizes the critical surveillance window [3]. Clinical examination combined with targeted imaging every 3–4 months during the first two years optimizes early detection.

The appearance of a new soft-tissue nodule not seen on immediate postoperative radiographs or previous interval surveillance imaging is suspicious for STR. Progressive growth on serial examinations distinguishes STR from static postoperative changes [1,5,32].

Heterotopic ossification, a common mimic, typically develops earlier (3–6 weeks postoperatively) and shows characteristic zonal maturation [4,35,39]. In contrast, STR ossification appears later and lacks a zonal architecture [5,40]. Postoperative scars or granulation tissue can appear as non-ossified, non-enhancing fibrous bands on MRI; these lesions do not enlarge over time and lack the aggressive enhancement or nodularity of STR. Occasionally, postoperative seromas or hematomas may persist, but they tend to decrease in size or resolve rather than grow [41].

When imaging findings remain equivocal, comparison with baseline postoperative studies is invaluable. Any new ossification or enhancement of the soft tissue mass warrants further evaluation. Image-guided biopsy should be considered when growth or concerning features are documented, as early intervention offers optimal results.

### 2.5 Pathology and molecular Signatures

Histologically, STR closely resembles primary GCTB, containing characteristic mononuclear stromal cells and osteoclast-like giant cells [6]. This similarity confirms true recurrence rather than reactive processes [1]. The identification of these features is essential for confirming the diagnosis of STR and differentiating it from other giant cell-rich lesions, such as soft-tissue giant cell tumors or malignant transformation [42].

Reactive metaplastic bone formation frequently accompanies STR, manifesting as peripheral or central ossification patterns [7].This osteogenic response is likely due to local tumor cell implantation and serves as a diagnostic hallmark [43].

Molecular analysis revealed retention of the H3F3A mutation (commonly G34W), a characteristic of GCTB. Immunohistochemical staining for the mutant H3.3 G34W protein provides valuable confirmation of recurrence [44].This molecular signature distinguishes STR from other giant cell-rich lesions or potential malignant transformation [45].

Malignant transformation remains rare in STR, typically associated with radiation exposure or multiple recurrences [46].When transformation occurs, high-grade sarcomatous features replace the typical GCTB morphology, often accompanied by additional genetic aberrations, including TP53 mutations [47,48]. The overall transformation risk remains low, supporting the predominantly benign nature of STR [32,49].

The RANK/RANKL pathway maintains its activity in STR lesions, similar to that in primary GCTB [34]. High RANKL expression in stromal cells promotes giant cell recruitment and activation. This pathway preservation provides a rationale for targeted therapies such as denosumab, although specific efficacy data for STR remain limited [50].

## 3 Management and outcomes

### 3.1 Surgical treatment

Although various management strategies may be appropriate for similar primary GCTB lesions, ranging from curettage to wide resection depending on anatomical location, functional considerations, and patient-specific factors, soft-tissue recurrence represents a different clinical setting. [51,52].

In STR, complete surgical excision with clear margins remains the preferred approach to minimize residual disease and reduce the likelihood of further recurrence. [18] While intralesional excision may be considered in select cases to preserve function, it carries a higher risk of repeat recurrence compared with margin-negative resection; thus, the choice of procedure should balance oncologic control with functional preservation, supported by close postoperative surveillance.[9,18].

**Fig. 3:**
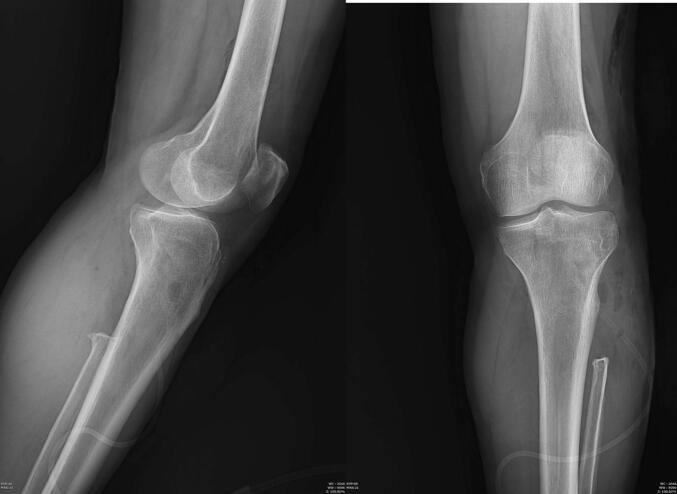
Postoperative plain radiograph following surgical resection of the proximal fibular lesion, confirming removal of the recurrent tumor.

When STR involves extensive soft tissue or previously treated bone, a more aggressive resection may be necessary [4,24]. En bloc removal of the affected bone segments may be required in selected cases. Although amputation remains rare, early detection and aggressive intervention typically permit limb salvage procedures with favorable outcomes [Fig. 3].

• **Imaging-Guided Surgical Planning**

Preoperative MRI with contrast enhancement defines the full extent of the disease and guides the surgical approach [6]. Fat-suppressed T2-weighted sequences can identify even small non-ossified components, whereas contrast-enhanced T1-weighted images delineate tumor-tissue interfaces [3,40]. This comprehensive imaging enables the planning of resections that encompass both visible tumors and microscopic extensions.

CT complements MRI by characterizing mineralized components and their relationships with bone or hardware [24]. When STR abuts critical structures, CT or MR angiography may clarify vessel involvement and inform reconstruction needs [40]. Intraoperative ultrasound can confirm the complete excision of superficial lesions [27]. This multimodal approach maximizes the likelihood of achieving negative margins while preserving the function.

### 3.2 Neoadjuvant/Adjuvant systemic therapy

Adjunctive systemic therapies, specifically denosumab and bisphosphonates, have shown efficacy in managing primary or unresectable GCTB; however, their role in STR remains unproven [36]. Denosumab, a monoclonal antibody targeting RANKL, reliably induces the disappearance of osteoclast-like giant cells and promotes peripheral sclerosis in intraosseous GCTB, thereby reducing morbidity [53,54]. Multiple case series have documented its use as a neoadjuvant treatment for grade II or III lesions to reduce tumor burden and create a calcified rim. However, retrospective reviews warn of a paradoxical increase in local recurrence after curettage following denosumab treatment, likely because the sclerotic border can mask residual tumor islands [55,56]. To date, no study has specifically evaluated the efficacy of denosumab against isolated STR mutations. [36] Consequently, using denosumab for STR must be approached with caution, although it may ossify a small soft tissue nidus and potentially make resection easier, suspending therapy without complete excision has precipitated rapid regrowth in intraosseous settings, suggesting a similar risk in STR [57,58].

Bisphosphonates, particularly zoledronic acid, exert anti‐osteoclastic effects by inhibiting farnesyl pyrophosphate synthase and inducing apoptosis in both osteoclast and tumor‐stromal cell lineages [59]. In metastatic or recurrent GCTB, prospective trials and retrospective series have shown that monthly zoledronic acid administration can stabilize pulmonary metastases and alleviate symptoms without significant progression over the follow-up periods [60,61]. However, in STR settings, where viable tumors reside exclusively in soft tissue, no prospective data have confirmed that bisphosphonates can eradicate or sufficiently suppress extraosseous stromal cells [62]. At best, they may slow the progression or temporarily control a small ossified STR focus and buy time until definitive wide excision [63].

Radiation therapy remains largely contraindicated for GCTB-related lesions because of the high risk (up to 25 %) of post-radiation sarcomatous transformation, particularly in younger patients [34,64]. Thus, its use is reserved only for exceptional circumstances, namely, unresectable STR in the axial skeleton (e.g., spinal or pelvic STR, where surgery would entail unacceptable morbidity). In rare cases, low-dose radiation protocols may be considered to achieve local control when systemic therapy and surgery are both unfeasible [65]. Overall, given the lack of high‐level evidence, systemic agents for STR should be viewed as adjunctive treatments rather than primary treatments. Denosumab can be considered a neoadjuvant treatment to induce ossification in challenging locations, and bisphosphonates may provide temporary stabilization; however, neither replaces the need for complete surgical excision when feasible.

### 3.3 Functional and oncologic outcomes

Most patients achieve excellent functional outcomes after STR excision. Recent series reported mean MSTS scores of 26.62 ± 4.21 (range 14–30), indicating preserved limb function after marginal or wide excision [36]. Similarly, in an earlier study by Xu et al., all six STR patients who underwent marginal resection expressed satisfaction with their surgical outcomes, achieving an average MSTS score of 28.0 ± 1.2 (range 26–29) at a mean follow-up of 13.4 ± 5.3 months[3.] These data confirm that when detected early and resected with adequate margins, STR rarely compromises mobility or daily activities.

Despite favorable functional results, multiple recurrences frequently occur. In the study by Cui et al., 61.3 % of patients experienced more than one STR event, with progressively shorter intervals between recurrences [36]. Notably, the interval between recurrences shortened with each subsequent event (first: 23.2 ± 26.1 months; second: 20.9 ± 18.1 months; third: 16.7 ± 12.5 months). This pattern suggests aggressive local behavior in certain cases and emphasizes the need for continued vigilance, even after an apparent cure.

Overall survival remains excellent, with STR rarely affecting the mortality rate. Among the 31 patients, only one death occurred, attributable to pulmonary metastasis rather than STR itself [36]. This finding aligns with broader GCTB data showing that local recurrence, while challenging, rarely compromises survival when appropriately managed [66].

### 3.4 Follow-Up Recommendations

Long-term surveillance after STR excision is essential because most recurrences, primary or additional, occur within the first 24 months, but can also present later (up to 30 months postoperatively) [67].Physical and radiological examinations of the surgical site are helpful in most cases. However, advanced imaging is helpful if recurrence is suspected. For example, Cui et la. During the initial two postoperative years, clinical examinations should be paired with high-resolution ultrasound of the operative bed every three–four months. The ability of ultrasound to detect small, non-ossified nodules makes it a sensitive and practical tool for early STR identification, particularly in superficial locations [36]. If any suspicious lesion is noted, such as a new hypoechoic nodule or interval growth, prompt MRI evaluation is indicated to confirm the extent of the lesion and guide the biopsy.

**Fig. 4:**
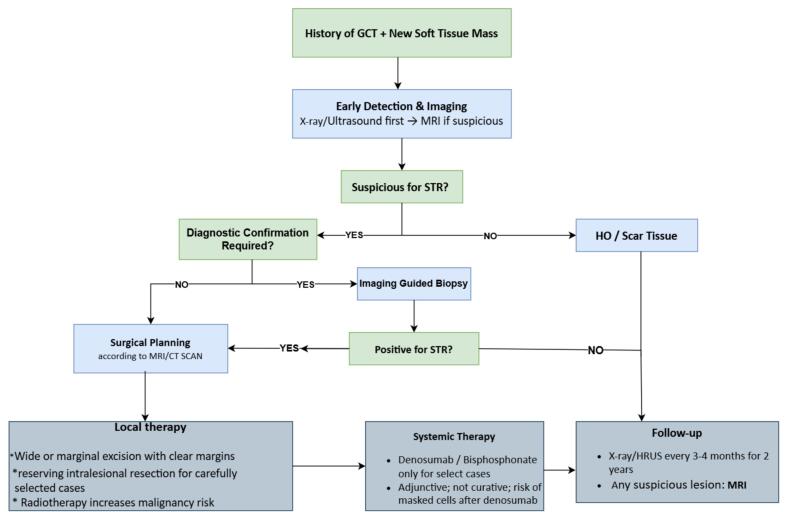
Proposed algorithm for the evaluation and management of soft-tissue recurrence (STR) in giant cell tumor of bone (GCTB).

Beyond 24 months, annual imaging should continue indefinitely, as late recurrences, although uncommon, have been documented for up to 30 months and occasionally beyond [4]. MRI of the surgical region is recommended to identify deep or infiltrative lesions that ultrasound may miss, and plain radiographs or CT scans should be obtained if ossification is suspected or if hardware from prior reconstructions obscures the soft tissue details. This prolonged follow-up period ensured that delayed STR events were detected early, allowing for timely intervention and preservation of optimal function. Table 1 summarizes the key domains of soft tissue recurrence in giant cell tumor of bone, including epidemiology, mechanisms, imaging characteristics, pathology, management strategies, and clinical outcomes [Table 1]. Fig. 4 presents a proposed treatment algorithm for soft-tissue recurrence in giant cell tumor of bone, outlining a structured approach to diagnosis and management [Fig. 4].

**Table 1:** Summary of epidemiology, mechanisms, imaging, pathology, management, and outcomes in soft tissue recurrence (STR) of giant cell tumor of bone (GCTB). References correspond to those in the main text.

Domain	Key Findings	Clinical Implication	References
Epidemiology & Risk	STR incidence ≈ 2–3 %; onset 6–12 months post-surgery; risk factors: curettage, pathological fracture, cortical breach, microscopic extension	High-risk patients need closer early surveillance	[3,12,13,15,16]
Pathogenesis	Mechanisms include iatrogenic seeding, hematoma contamination, microscopic invasion, limited effect of adjuvants	Preventive strategies: meticulous curettage, excision of hematoma/pseudocapsule, wound protection	[4,7,22,25,26,33]
Imaging	Three patterns: (I) peripheral rim ossification, (II) central nodular calcification, (III) non-ossified; MRI defines extent; ultrasound detects early superficial nodules	Early differentiation from HO or scar tissue	[3,5,6,30,36,37]
Pathology & Molecular	STR histology mirrors GCTB with osteogenic metaplasia; retains H3F3A (G34W) mutation	Confirms recurrence and excludes mimics	[6,7,42,44,45]
Management	Complete surgical excision is standard; denosumab and bisphosphonates investigational; radiotherapy contraindicated	Surgery definitive; systemic therapy adjunctive only	[9,18,36,53,59,62]
Outcomes & Follow-up	Good function (MSTS > 26); ∼60 % risk of multiple recurrences; overall survival excellent	Close follow-up every 3–4 months for 2 years, then annually	[3,12,36,65]

## 4 Discussion

Our narrative synthesis confirms that STR in GCTB remains rare, with approximately 2 %-3 % of cases when rigorous imaging (MRI and high‐resolution ultrasound) is used to detect non-ossified lesions [3,36]. Most STRs were present between 5 and 17 months postoperatively (median, 11 months), with > 60 % occurring within the first year. Imaging typically reveals one of three patterns: peripheral “eggshell” ossification (Type I), central calcified nodules (Type II), or purely soft‐tissue masses (Type III) [6]. Ultrasound sensitively detects small, non-ossified STRs before radiographic changes, whereas MRI defines the full lesion extent, including infiltrative strands masked by ossified rims [36]. Early detection allows for marginal or wide excision to achieve R0 margins, yielding excellent functional outcomes and durable local control. However, up to 60 % of patients experience ≥ 2 STR events, indicating aggressive local behavior in a subset.

Although imaging remains the primary modality for detecting recurrence in GCTB, several studies have explored whether systemic inflammatory markers may assist in predicting recurrence or prognosis. Inflammatory indices such as neutrophil-to-lymphocyte ratio (NLR), platelet-to-lymphocyte ratio (PLR), lymphocyte-to-monocyte ratio (LMR), prognostic nutritional index (PNI), and modified Glasgow Prognostic Score (mGPS) have been evaluated. [68,69] A recent study reported no significant association between these markers and local recurrence in GCTB, suggesting limited utility in routine clinical practice. Conversely, prior research on spinal GCTB demonstrated that elevated NLR and PLR, and reduced LMR and albumin-to-globulin ratio (AGR), may correlate with poorer disease-free survival.[68,69].

To date, the role of serum inflammatory biomarkers in predicting recurrence or clinical outcome in GCTB remains uncertain. Preliminary studies have produced conflicting results, with one report demonstrating no significant association between systemic inflammatory parameters and local recurrence, while another analysis in spinal GCTB suggested potential prognostic value for NLR, PLR, LMR, and AGR in stratifying recurrence risk. Given these heterogeneous findings, inflammatory markers should currently be considered exploratory rather than clinically validated tools in GCTB surveillance.[68,69] Future prospective studies with larger patient cohorts are warranted to clarify whether these laboratory indices may complement imaging-based.

The pathogenesis of STR reflects overlapping mechanisms. Iatrogenic seeding during intralesional curettage can occur as uncontained curettage can displace viable stromal and giant cells into adjacent tissues [4]. Pathological fractures triple to quadruple the risk (hazard ratio ≈ 3.5; p < 0.01) by permitting tumor-laden hematoma to seed soft tissues, particularly if fracture hematoma or pseudocapsule remains postoperatively [15]. Approximately 25 % of STR cases demonstrate microscopic soft-tissue invasion that is not visible on preoperative MRI, allowing small nests of neoplastic cells to persist outside the bone.[25,67] Reactive pseudocapsules can conceal these foci of inflammation. Additionally, local adjuvants (phenol, cryotherapy, and alcohol) and PMMA cement penetrate only 2–3 mm beyond the cavity wall; any microscopic cortical breach enables escaped cells to avoid cytotoxic effects and later proliferate into STR [70].

Therefore, meticulous preventive measures are essential to avoid such complications. Whenever feasible, en bloc excision minimizes the STR risk (0–0.5 % in large series). If curettage is necessary, strict containment, dedicated irrigation basins, separate instruments, wound protectors, thorough excision of the fracture hematoma, pseudocapsule, and any MRI-identified soft tissue extension are critical [8]. Adjuvant use should account for their limited range, and cortical defects must be extended to ensure that the chemical or thermal effect reaches the displaced cells. Postoperatively, clinical findings and targeted imaging surveillance every 3 months for the first 2 years can identify nascent STRs, facilitating early re-excision before extensive soft-tissue infiltration occurs [67].

Systemic therapies, such as denosumab and bisphosphonates, remain unproven for STR. Denosumab induces sclerosis in intraosseous GCTB but may mask viable cells behind sclerotic rims, with no data on isolated STR efficacy [55]. Bisphosphonates stabilize metastatic or recurrent disease in case reports, yet no evidence supports their use in extraosseous STR [60]. Radiotherapy carries up to a 25 % risk of malignant transformation and is reserved for unresectable axial STR [65]. Consequently, surgical excision remains the cornerstone of treatment, with systemic agents used only to temporize tumors when resection is not immediately feasible.

Although STR rarely compromises overall survival, five-year survival exceeds 95 % when managed appropriately, it frequently recurs locally and may herald occult metastases. In a recent cohort study, 12.9 % of patients displayed pulmonary nodules, with one death attributable to metastasis rather than STR. Multiple recurrences shorten the latency intervals (23.2 to 16.7 months), reflecting aggressive biology [36]. Therefore, we recommend quarterly clinical examinations and, if needed, ultrasound or MRI for two years, followed by 6 months for the third year and then annually, complemented by purposeful clinicoradiologic follow-up to detect deep or infiltrative lesions, given documented late recurrences up to 30 months. Future research should focus on multicenter registries, prospective trials of neoadjuvant systemic agents, molecular predictors (e.g., circulating H3F3A DNA [45]), and advanced imaging (contrast-enhanced ultrasound and PET/MRI) to refine risk stratification, early detection, and prevention strategies.

## 5 Conclusion

Soft-tissue recurrence (STR) of giant cell tumors of the bone (GCTB) is an uncommon but distinct and clinically relevant condition. Although often under-recognized, STR is typically benign, non-infiltrative, and curable with marginal excision, yielding excellent functional outcomes. Accurate diagnosis relies on awareness of imaging patterns, particularly non-ossified lesions detectable only by MRI, and recognition of key risk factors such as curettage and pathological fractures. Although STR shares molecular features with primary GCTB, no biomarkers currently predict its behavior or guide the therapy. Future efforts should focus on establishing surveillance protocols, validating molecular targets, and clarifying the roles of systemic therapy. A multidisciplinary, risk-adapted approach is essential to optimize outcomes in this rare but important subset of GCTB recurrences.

## CRediT authorship contribution statement

**Khodamorad Jamshidi:** Writing – review & editing, Writing – original draft, Methodology, Conceptualization. **Hamed Naghizadeh:** Writing – review & editing, Writing – original draft, Methodology, Conceptualization. **Sadegh Saberi:** Writing – review & editing, Writing – original draft. **Farshad Zand Rahimi:** Writing – review & editing, Writing – original draft. **Aidin Arabzadeh:** Writing – review & editing, Writing – original draft. **Seyyed Saeed Khabiri:** Writing – review & editing, Writing – original draft, Methodology, Conceptualization.

## Declaration of competing interest

The authors declare that they have no known competing financial interests or personal relationships that could have appeared to influence the work reported in this paper.

## References

[1] Turcotte R.E. (Jan 2006). Giant cell tumor of bone. Orthop. Clin. North Am..

[2] Errani C., Ruggieri P., Asenzio M.A. (Feb 2010). Giant cell tumor of the extremity: a review of 349 cases from a single institution. Cancer Treat. Rev..

[3] Xu L., Jin J., Hu A. (Nov 2017). Soft tissue recurrence of giant cell tumor of the bone: Prevalence and radiographic features. Journal of Bone Oncology..

[4] Yang Q., Wang L., Yang Z., Li X., Meng B. (2009). Li JJTC-GJoCO. Soft Tissue Recurrence of Giant Cell Tumor of Bone: A Report of Two Cases and Literature Review..

[5] Ehara S., Nishida J., Abe M., Kawata Y., Saitoh H. (1992). Kattapuram SVJCi. Ossified Soft Tissue Recurrence of Giant Cell Tumor of Bone..

[6] Park S.-Y., Lee M.H., Lee J.S., Song J.S. (2014). Chung HWJSr. Ossified Soft Tissue Recurrence of Giant Cell Tumor of the Bone: Four Case Reports with Follow-up Radiographs, CT, Ultrasound, and MR Images..

[7] Teot L.A., O'Keefe R.J., Rosier R.N., O'Connell J.X., Fox E.J., Hicks DGJHp. (1996). Extraosseous Primary and Recurrent Giant Cell Tumors: Transforming Growth Factor-Β1 and-Β2 Expression May Explain Metaplastic Bone Formation..

[8] Jamshidi K., Zandrahimi F., Haji Agha Bozorgi M. (2021). Extended curettage versus en bloc resection for the treatment of grade 3 giant cell tumour of the knee with pathologic fracture: a retrospective study. Int. Orthop..

[9] Pitsilos C., Givissis P., Papadopoulos P., Chalidis B.J.C. (2023). Treatment of Recurrent Giant Cell Tumor of Bones: a Systematic Review..

[10] Wong G., Greenhalgh T., Westhorp G., Buckingham J. (2013). Pawson RJJoAN. RAMESES Publication Standards: Meta-Narrative Reviews..

[11] Von Borstel D., Taguibao A. (2017). R, a Strle N, E Burns JJSr. Giant Cell Tumor of the Bone: Aggressive Case Initially Treated with Denosumab and Intralesional Surgery..

[12] Pai S.N. (2021). Rajappa SJJoOCR. Recurrent Giant Cell Tumor of Distal Radius with Pulmonary Metastasis: a Case Report..

[13] AbdelKawi N.M., Abed Y.Y., El-negery A.A., Kotb S.Z.M.J.C.O.P. (2023). Risk Factors for Local Recurrence of Giant Cell Tumor of Bone of the Extremities: a Retrospective Study..

[14] Hasan O., Ali M., Mustafa M., Ali A., Umer MJAoM, (2019). Surgery. Treatment and Recurrence of Giant Cell Tumors of Bone–a Retrospective Cohort from a Developing Country..

[15] Hu P., Zhao L., Zhang H. (2016). Recurrence Rates and Risk Factors for Primary Giant Cell Tumors around the Knee: a Multicentre Retrospective Study in China..

[16] Tsukamoto S., Mavrogenis A.F., Hindiskere S. (2022). Outcome of Reoperation for Local Recurrence following En Bloc Resection for Bone Giant Cell Tumor of the Extremity..

[17] O'Donnell R.J., Springfield D.S., Motwani H.K., Ready J.E., Gebhardt M.C., Mankin H.J.J.J. (1994). Recurrence of Giant-Cell Tumors of the Long Bones after Curettage and Packing with Cement..

[18] He Y., Zhang J., Ding XJLrm. (2017). Prognosis of Local Recurrence in Giant Cell Tumour of Bone: What Can We Do?.

[19] Wang H., Wan N., Hu Y.J.I.O. (2012). Giant Cell Tumour of Bone: a New Evaluating System Is Necessary..

[20] Lin X., Liu J., Xu M.J.T.C.R. (2021). The Prognosis of Giant Cell Tumor of Bone and the Vital Risk Factors That Affect Its Postoperative Recurrence: a Meta-Analysis..

[21] Ngan K.-W., Chuang W.-Y. (2008). Yeh C-JJP-JotR. Soft Tissue Recurrence of Sacral Giant Cell Tumour of Bone as an Intra-Abdominal Mass: an Unusual Presentation..

[22] Chen L., Ding X.-Y., Wang C.-S. (2014). In-Depth Analysis of Local Recurrence of Giant Cell Tumour of Bone with Soft Tissue Extension after Intralesional Curettage..

[23] Younis A., Aziz S.A. (2004). El Shahawy MJJotENCI. Surgical Management for Giant Cell Tumor of Bones..

[24] Georgiev G.P., Slavchev S., Dimitrova I.N., Landzhov BJJoC, Investigations E. (2014). Giant Cell Tumor of Bone: Current Review of Morphological, Clinical, Radiological, and Therapeutic Characteristics..

[25] Prosser G, Baloch K, Tillman R, Carter S, Grimer RJCO, Research® R (2005). Does Curettage without Adjuvant Therapy Provide Low Recurrence Rates in Giant-Cell Tumors of Bone?.

[26] Zacharia B., Pai P.K. (2020). Paul MJIJoSO. Clinical and Radiological Profile of Ten Interesting Though Rare Presentations of Giant Cell Tumor of Bone..

[27] Akaike K., Suehara Y., Takagi T., Kaneko K. (2014). Saito TJSr. An Eggshell-like Mineralized Recurrent Lesion in the Popliteal Region after Treatment of Giant Cell Tumor of the Bone with Denosumab..

[28] Lee F.-Y.-I., Montgomery M., Hazan E.J., Keel S.B., Mankin H.J., Kattapuram S.J.J. (1999). Recurrent giant-cell tumor presenting as a soft-tissue mass. A Report of Four Cases..

[29] Szendröi MJTJoB, Volume JSB. (2004). Giant-Cell Tumour of Bone..

[30] Pereira H.M., Marchiori E. (2014). Severo AJJoMI, Oncology R. Magnetic Resonance Imaging Aspects of Giant-Cell Tumours of Bone..

[31] Algawahmed H., Turcotte R., Farrokhyar F., Ghert M.J.S. (2010). High-Speed Burring with and without the Use of Surgical Adjuvants in the Intralesional Management of Giant Cell Tumor of Bone: A Systematic Review and Meta-Analysis..

[32] van der Heijden L., van de Sande M.A., Dijkstra P.D. (Aug 2012). Soft tissue extension increases the risk of local recurrence after curettage with adjuvants for giant-cell tumor of the long bones. Acta Orthop..

[33] Mavrogenis A.F., Igoumenou V.G., Megaloikonomos P.D., Panagopoulos G.N., Papagelopoulos P.J. (2017). Soucacos PNJS-j. Giant Cell Tumor of Bone Revisited..

[34] van der Heijden L., Dijkstra P.D., van de Sande M.A. (May 2014). The clinical approach toward giant cell tumor of bone. Oncologist.

[35] Cooper K., Beabout J., Dahlin D.J.R. (1984). Giant Cell Tumor: Ossification in Soft-Tissue Implants..

[36] Cui L., Sun Y., Jin T., Fan D., Liu W.J.D.O. (2024). Giant Cell Tumor of Bone at Distal Radius Suffered More Soft Tissue Recurrence and Ultrasonography Is Effective to Detect the Soft Tissue Recurrence..

[37] Kwee R.M. (2019). Kwee TCJSR. Calcified or Ossified Benign Soft Tissue Lesions That May Simulate Malignancy..

[38] Crompton S., Hughes D., Musson R.J.C.R. (2025). A Pictorial Review of Osseous and Cartilaginous Soft Tissue Tumours..

[39] McCarthy E.F., Sundaram M. (Oct 2005). Heterotopic ossification: a review. Skeletal Radiol..

[40] Murphey M.D., Nomikos G.C., Flemming D.J., Gannon F.H., Temple H.T., Kransdorf M.J. (2001). From the archives of AFIP. Imaging of giant cell tumor and giant cell reparative granuloma of bone: radiologic-pathologic correlation. *Radiographics : a review publication of the Radiological Society of North America, Inc*. Sep-Oct.

[41] Kim S.Y., Park J.S., Ryu K.N., Jin W., Park S.Y. (2011). Various tumor-mimicking lesions in the musculoskeletal system: causes and diagnostic approach. Korean Journal of Radiology. Mar-Apr.

[42] Chakarun C.J., Forrester D.M., Gottsegen C.J., Patel D.B., White E.A., Matcuk G.R. (2013). Giant cell tumor of bone: review, mimics, and new developments in treatment. *Radiographics : a review publication of the Radiological Society of North America, Inc*. Jan-Feb.

[43] James I.E., Dodds R.A., Olivera D.L., Nuttall M.E. (1996). Gowen MJJoB, Research M. Human Osteoclastoma-Derived Stromal Cells: Correlation of the Ability to Form Mineralized Nodules in Vitro with Formation of Bone in Vivo..

[44] Khazaei S., De Jay N., Deshmukh S. (2020). H3. 3 G34W Promotes Growth and Impedes Differentiation of Osteoblast-like Mesenchymal Progenitors in Giant Cell Tumor of Bone..

[45] Luo Y., Tang J., Huang J. (2023). Diagnostic Value of H3F3A Mutation and Clinicopathological Features of Giant Cell Tumours in Non-Long Bones..

[46] Palmerini E., Picci P., Reichardt P. (2019). Downey GJTicr, treatment. Malignancy in Giant Cell Tumor of Bone: a Review of the Literature..

[47] Taqi M. (2024). ul Rasool H, Zaka Haider M, Al Muderis MJD. Significance of Biogenetic Markers in Giant Cell Tumor Differentiation and Prognosis: A Narrative Review..

[48] Yang M., Wang F., Liang H. (2023). Single-Cell RNA Sequencing Reveals Distinct Immune Cell Subsets in Phalangeal and Soft-Tissue Recurrence of Giant Cell Tumor of Bone..

[49] Vari S., Riva F., Onesti C.E. (2022). Malignant Transformation of Giant Cell Tumour of Bone: a Review of Literature and the Experience of a Referral Centre..

[50] Palmerini E., Seeger L.L., Gambarotti M. (2021). Malignancy in Giant Cell Tumor of Bone: Analysis of an Open-Label Phase 2 Study of Denosumab..

[51] Saberi S., Naghizadeh H., Heydari S., Poorghasem E., Bayati M., Khabiri S.S. (2025). Optimizing proximal fibula tumor resection:“The open door technique” for better surgical access and outcomes-a case series. Int. J. Surg. Case Rep..

[52] Hashimoto K., Nishimura S., Goto K. (2025). Giant Cell Tumor of the Proximal Fibula in a 15-year-old Female: a Review and Case Report. In Vivo.

[53] Thomas D., Henshaw R., Skubitz K. (2010). Denosumab in Patients with Giant-Cell Tumour of Bone: an Open-Label, Phase 2 Study..

[54] Yue J., Sun W. (2022). Li SJJoBO. Denosumab versus Zoledronic Acid in Cases of Surgically Unsalvageable Giant Cell Tumor of Bone: A Randomized Clinical Trial..

[55] Agarwal MG, Gundavda MK, Gupta R, Reddy RJCO, Research® R (2018). Does denosumab change the giant cell tumor treatment strategy?. Lessons Learned from Early Experience..

[56] Parmeggiani A., Miceli M., Errani C., Facchini G.J.C. (2021). State of the Art and New Concepts in Giant Cell Tumor of Bone: Imaging Features and Tumor Characteristics..

[57] Niu X., Yang Y., Wong K.C., Huang Z., Ding Y., Zhang WJJoot. (2019). Giant Cell Tumour of the Bone Treated with Denosumab: How Has the Blood Supply and Oncological Prognosis of the Tumour Changed?.

[58] Chawla S., Henshaw R., Seeger L. (2013). Safety and Efficacy of Denosumab for Adults and Skeletally Mature Adolescents with Giant Cell Tumour of Bone: Interim Analysis of an Open-Label, Parallel-Group, Phase 2 Study..

[59] Yokoyama T., Mizuguchi M., Ostermann A. (2015). Protonation State and Hydration of Bisphosphonate Bound to Farnesyl Pyrophosphate Synthase..

[60] Dubey S., Rastogi S., Sampath V., Khan S.A. (2019). Kumar AJJoCO, Trauma. Role of Intravenous Zoledronic Acid in Management of Giant Cell Tumor of Bone-a Prospective, Randomized, Clinical, Radiological and Electron Microscopic Analysis..

[61] Balke M., Campanacci L., Gebert C. (2010). Bisphosphonate Treatment of Aggressive Primary, Recurrent and Metastatic Giant Cell Tumour of Bone..

[62] Chang S.S., Suratwala S.J., Jung K.M. (2004). Bisphosphonates May Reduce Recurrence in Giant Cell Tumor by Inducing Apoptosis..

[63] López-Pousa A., Broto J.M., Garrido T., Vázquez J.J.C., Oncology T. (2015). Giant Cell Tumour of Bone: New Treatments in Development..

[64] Shi W., Indelicato D.J., Reith J. (2013). Radiotherapy in the Management of Giant Cell Tumor of Bone..

[65] Chen W., Yan Z. (2020). Tirumala VJJoOR. Malignant Giant Cell Tumor of Bone or Soft Tissue Treated by Surgery with or without Radiotherapy..

[66] Yayan JJAiB. (2019,). Increased Risk of Lung Metastases in Patients with Giant Cell Bone Tumors: a Systematic Review..

[67] Balke M., Ahrens H., Streitbuerger A. (2009). Treatment Options for Recurrent Giant Cell Tumors of Bone..

[68] Tsukamoto S., Mavrogenis A.F., Alvarado R.A. (2023). Association between Inflammatory Markers and Local Recurrence in patients with Giant Cell Tumor of Bone: a Preliminary result. Curr. Oncol..

[69] Li J., Li B., Zhou P. (2017). Nomograms for prognostic factors of spinal giant cell tumor combining traditional clinical characteristics with inflammatory biomarkers after gross total resection. Oncotarget.

[70] Wang Y., Tian Q., Wu C., Li H., Li J. (2021). Feng YJFiS. Management of the Cavity after Removal of Giant Cell Tumor of the Bone..

